# Decaffeinated Green Coffee Bean Extract Attenuates Diet-Induced Obesity and Insulin Resistance in Mice

**DOI:** 10.1155/2014/718379

**Published:** 2014-04-10

**Authors:** Su Jin Song, Sena Choi, Taesun Park

**Affiliations:** Department of Food and Nutrition, Brain Korea 21 PLUS Project, Yonsei University, 50 Yonsei-ro, Seodaemun-gu, Seoul 120-749, Republic of Korea

## Abstract

This study investigated whether decaffeinated green coffee bean extract prevents obesity and improves insulin resistance and elucidated its mechanism of action. Male C57BL/6N mice (*N* = 48) were divided into six dietary groups: chow diet, HFD, HFD-supplemented with 0.1%, 0.3%, and 0.9% decaffeinated green coffee bean extract, and 0.15% 5-caffeoylquinic acid. Based on the reduction in HFD-induced body weight gain and increments in plasma lipids, glucose, and insulin levels, the minimum effective dose of green coffee bean extract appears to be 0.3%. Green coffee bean extract resulted in downregulation of genes involved in WNT10b- and galanin-mediated adipogenesis and TLR4-mediated proinflammatory pathway and stimulation of GLUT4 translocation to the plasma membrane in white adipose tissue. Taken together, decaffeinated green coffee bean extract appeared to reverse HFD-induced fat accumulation and insulin resistance by downregulating the genes involved in adipogenesis and inflammation in visceral adipose tissue.

## 1. Introduction

Coffee is one of the most widely consumed beverages in the world, and therefore the potential health consequences of coffee consumption are of great public interest. Heavy coffee drinking may result in sleep disorders, hypokalemia, and cardiac arrhythmias [1–4]. At the same time, several epidemiologic studies have reported that the risk of Parkinson's disease, Alzheimer's disease, and certain types of cancer is reduced in regular coffee consumers [5]. In addition, coffee has recently received scientific attention as current epidemiologic and* in vivo* studies have revealed its health benefits against obesity and metabolic disorders, especially type 2 diabetes [6–10]. These health advantages are mostly derived from chlorogenic acids contained in coffee beans [11–14].

Adipogenesis is a process of mesenchymal precursor cells differentiating into adipocytes where peroxisome proliferator-activated receptor *γ*2 (PPAR*γ*2) and CCAAT/enhancer-binding protein *α* (C/EBP*α*) are the master transcriptional regulators [1, 15]. Downstream targets for PPAR*γ*2 include adipocyte lipid binding protein (aP2), cluster of differentiation 36 (CD36), lipoprotein lipase (LPL), and fatty acid synthase (FAS), which together induce lipid accumulation and metabolism. Thus, inactivation of these adipogenic regulators may be a novel way to suppress adipogenesis and ultimately prevent obesity.

Another critical aspect of adipose tissue is that it serves as an endocrine organ, releasing biologically active adipokines. Toll-like receptor (TLR) 2 and TLR4 induce the expression of a large number of proinflammatory target genes. It was recently discovered that TLR4 can sense free fatty acids (FFAs) engaging proinflammatory pathways that lead to secretion of cytokines [16]. Furthermore, several studies have demonstrated a causative relationship between inflammation and insulin resistance [17]. JNK, especially, serves as a main mediator that leads to insulin resistance by impairing GLUT4 translocation. Thus, reducing the FFA level in blood and peripheral tissues such as the adipose tissue and muscles might result in therapeutic effects against obesity by attenuating not only adipogenesis but also inflammation and insulin resistance.

Raw coffee beans are rich in chlorogenic acids and caffeine, and their contents in coffee beans are significantly decreased during the roasting and decaffeination processes [18]. Green coffee bean extract used in the present study is prepared from decaffeinated and unroasted coffee beans, making it a novel source of chlorogenic acids and eliminating the possible side effects of caffeine [1–3]. There is no report on toxicological studies on green coffee bean extract. In a clinical trial, decaffeinated green coffee bean extract induced weight loss in overweight volunteers, provided with 400 mg/day for 60 days [19]. Yet, further investigation on toxic dose of green coffee bean extract is required in animal models. Cho et al. revealed that 5-caffeoylquinic acid (CQA), a representative chlorogenic acid in green coffee beans, exhibits antiobesity properties in mice fed a HFD [12]. Another study reported that decaffeinated green coffee bean extract, delivered through drinking water for 20 weeks, significantly improved HFD-induced insulin resistance in mice; however, the dose delivered to mice was not clearly indicated and its molecular mechanism on improving insulin sensitivity has not been examined [10]. Also, the antiobesity effect of decaffeinated green coffee bean extract has not yet been reported in HFD-induced obese mice. Therefore, the aims of this study were to investigate whether decaffeinated green coffee bean extract exerts protective effects against visceral obesity and insulin resistance in mice fed a HFD and to evaluate whether these effects are derived from 5-CQA. Furthermore, we explored the potential molecular mechanisms of the health benefits of decaffeinated green coffee bean extract, focusing on the gene expression involved in adipogenesis and insulin resistance in white adipose tissue (WAT).

## 2. Materials and Methods

### 2.1. Extraction and HPLC Analysis of Decaffeinated Green Coffee Bean Extract

The decaffeinated green coffee bean extract utilized for this study was provided by Naturex Inc. (Avignon, France) under the trade name Svetol. Svetol was obtained by extracting decaffeinated raw green coffee (*Coffea canephora robusta*) beans with 30% ethanol at 70°C for 2 h. To determine 5-CQA (IUPAC numbering) content in decaffeinated green coffee bean extract, HPLC analysis was performed via a Supelco C18 column (250 × 4.6 mm, 5 *μ*m inner diameter) at 40°C with a flow rate of 1.4 mL/min using a gradient mobile phase composed of water (A) and acetonitrile (B). The mobile phase was 95 : 5 mixture of components A and B as the initial condition of the chromatography; the sample injection volume was 2 *μ*L. The absorption spectrum of 5-CQA was monitored at 330 nm using the photodiode array detector.

### 2.2. Animal Care and Experimental Protocol

Forty-eight male C57BL/6N mice (Orient, Gyeonggi-do, Republic of Korea) were housed in standard cages and placed in a room where the temperature was maintained at 23 ± 1°C, relative humidity at 50 ± 1%, and the light at a 12 h light/dark cycle. During a 1-week acclimatization period, all mice consumed a commercial diet and tap water* ad libitum*. Afterwards, they were divided into six weight-matched groups (*n* = 8): the chow diet (CD), high-fat diet (HFD), 0.1%, 0.3%, and 0.9% green coffee bean extract-supplemented diet (GCD), and 0.15% 5-CQA-supplemented diet (CQD) groups (Sigma, MO, USA). The HFD was composed of 200 g of fat/kg (170 g of lard plus 30 g of corn oil) and 1% (w/w) cholesterol. The GCD was identical to the HFD, except that it included 0.1%, 0.3%, or 0.9% green coffee bean extract. The CQD was also identical to the HFD except that it contained 0.15% 5-CQA. The diets were given in the form of pellets for eleven weeks.

Food intake of the mice was recorded daily and their body weights were measured weekly during the feeding period. At the end of the experimental period, the animals were anesthetized with ether following a 12 h fasting period. Blood samples were drawn from the abdominal aorta into an EDTA-coated tube, and plasma samples were obtained by centrifugation at 1,000 ×g for 15 min at 4°C. Visceral fat pads from four different regions (epididymal, perirenal, mesenteric, and retroperitoneal regions) were excised, rinsed with phosphate-buffered saline (PBS), and stored at −80°C until analysis. All animal experiments adhered to the Korean Food and Drug Administration (KFDA) guidelines. The protocols were reviewed and approved by the Institutional Animal Care and Use Committee (IACUC) of the Yonsei Laboratory Animal Research Center (YLARC) (Permit no. 2013-0104). All mice were maintained in the specific pathogen-free facility of the YLARC.

### 2.3. Histological Analysis

The epididymal fat pads were fixed in neutral buffered formalin and embedded in paraffin, sectioned at thicknesses of 5 *μ*m. The tissue sections were stained with hematoxylin and eosin (H&E).

### 2.4. Biochemical Analysis

The plasma concentrations of triglycerides (TG), FFA, total cholesterol (TC), and glucose were measured enzymatically using commercial kits (Bio-Clinical System, Gyeonggi-do, Republic of Korea). Plasma leptin, adiponectin, interleukin-6 (IL-6), monocyte chemoattractant protein-1 (MCP-1), and insulin levels were analyzed using an ELISA kit (Millipore, MA, USA). The homeostasis model assessment of basal insulin resistance (HOMA-IR) was used to calculate an index from the product of the fasting concentrations of plasma glucose (mmol/L) and insulin (pmol/L) divided by 22.5. Lower HOMA-IR values indicate greater insulin sensitivity and higher HOMA-IR values indicate insulin resistance.

### 2.5. Oral Glucose Tolerance Test

An oral glucose tolerance test (OGTT; gavage with 2 g glucose/10 mL per kg body weight) was performed 2 weeks before the end of the treatment on 18 h fasted mice by administering glucose orally. Blood was collected from the tail vein at 0, 15, 30, 60, 90, and 120 min following glucose administration to determine blood glucose.

### 2.6. Semiquantitative Reverse Transcriptase Polymerase Chain Reaction (RT-PCR)

Total RNA was isolated from the epididymal adipose tissue of each mouse with Trizol (Invitrogen, CA, USA). Of the total RNA, 4 *μ*L was reverse-transcribed to cDNA using the Superscript II kit (Invitrogen) according to the manufacturer's instructions.  Table 1 shows the forward (F) and reverse (R) primer sequences. The PCR procedure was designed as follows: 10 min at 94°C, 30–35 cycles at 94°C for 30 s, 55°C for 30 s, 72°C for 1 min, and 10 min of incubation at 72°C. Next, 4 *μ*L of each PCR reaction mixture was mixed with 1 *μ*L of 6-fold-concentrated loading buffer and then loaded onto a 2% agarose gel containing ethidium bromide. The mRNA levels were normalized to the glyceraldehyde-3-phosphate dehydrogenase (GAPDH) mRNA levels, which were used as an internal control.

### 2.7. Western Blot Analysis

The epididymal adipose tissues of each mouse were homogenized in an extraction buffer containing 100 mM Tris-HCl, pH 7.4, 5 mM EDTA, 50 mM sodium pyrophosphate, 50 mM NaF, 100 mM orthovanadate, 1% Triton X-100, 1 mM phenylmethanesulfonyl fluoride, 2 *μ*g/mL aprotinin, 1 *μ*g/mL pepstatin A, and 1 *μ*g/mL leupeptin. The tissue homogenates were centrifuged at 1,300 ×g for 20 min at 4°C. The protein concentrations of the tissue extracts were measured via Bradford assay (Bio-Rad, CA, USA). The protein samples were separated by SDS-PAGE and electrophoretically transferred to nitrocellulose membranes (Amersham, Buckinghamshire, UK). The samples were incubated overnight and hybridized with primary antibodies (diluted 1 : 1,000) at 4°C. Antibodies to the following proteins were purchased from the indicated sources: *β*-catenin, *β*-actin (Santa Cruz Biotechnology, CA, USA), c-Jun N-terminal kinase (JNK), p-JNK (Tyr183), insulin receptor substrate (IRS), p-IRS (Ser307), protein kinase B (AKT), p-AKT (Thr308), GLUT4, and GAPDH (Cell Signaling Technology, MA, USA). The membranes were incubated with the corresponding secondary antibody. Next, immunoreactive signals were detected via a chemiluminescent detection system (Amersham, Buckinghamshire, UK) and quantified using Quantity One analysis software (Bio-Rad).

### 2.8. Statistical Analysis

The data on body weight gain, plasma biochemistries, and adipocyte diameter is expressed as the mean ± SEM of 8 mice. The RT-PCR and Western data are means from *n* = 8 ± SEM of three independent experiments (*n* = 2, 3 per experiment) for each group. Data were analyzed by one-way analysis of variance (ANOVA), followed by Duncan's multiple range tests. *P* values < 0.05 were considered statistically significant.

## 3. Results

### 3.1. HPLC Analysis of Decaffeinated Green Coffee Bean Extract

**Figure 1:**
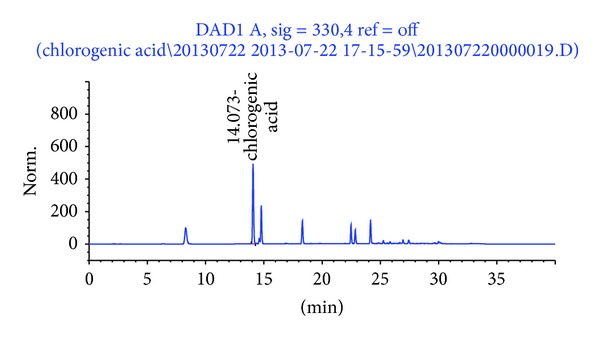
The HPLC chromatogram of decaffeinated green coffee bean extract. The peak was assigned based on the isolation of 5-CQA.

The extraction yield of decaffeinated green coffee beans was 15%. The HPLC analysis (Figure 1) revealed that decaffeinated green coffee bean extract (Svetol) contained 16.4% 5-CQA.

### 3.2. Body and Visceral Fat-Pad Weights

**Figure 2:**
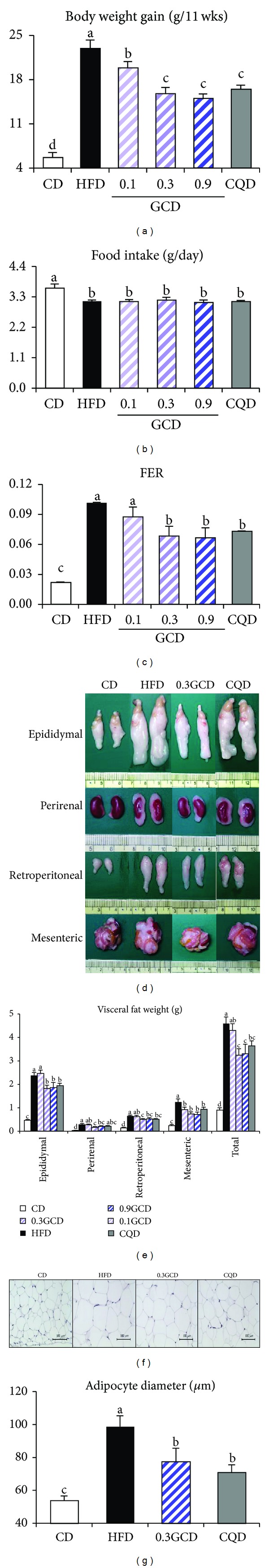
Effects of green coffee bean extract and 5-CQA supplementation on body weight gain, food efficiency ratio (FER), and visceral fat-pad weights of mice fed a HFD. Mice were fed the experimental diets for 11 weeks. (a) Body weight gain, (b) food intake, (c) FER, ((d), (e)) visceral fat-pad weights, (f) representative pictures of H&E-stained fat cells from mice epididymal adipose tissue (×100), and (g) densitometric analysis of adipocyte diameter in epididymal tissue. Data represent mean ± SEM, *n* = 8. Mean values indicated with different letters indicate statistical significance (*P* < 0.05). FER = body weight gain for experimental period (g)/food intake for experimental period (g).

After 11 weeks of experimental feeding, the final body weight gain was dose-dependently decreased in the 0.1GCD and 0.3GCD groups (Figure 2(a)). Food intake did not differ among experimental groups during the 11-week feeding period (Figure 2(b)), and the food efficiency ratio (FER) was significantly decreased in mice fed the 0.3GCD when compared with mice fed the HFD (Figure 2(c)). The total visceral fat-pad weight of mice fed the HFD was reduced when the mice were supplemented with 0.3% green coffee bean extract (Figures 2(d) and 2(e)). No further reduction in body weight gain and visceral fat-pad weight was noted in the 0.9GCD group. Moreover, 0.3% green coffee bean extract decreased body weight gain and visceral adiposity as much as 0.15% 5-CQA did. Based on the results above, 0.3% appears to be the minimum effective dose at which green coffee bean extract reduces body weight gain and visceral fat-pad weight. Therefore, the histological analysis of epididymal adipose tissue sections by H&E staining was done with the 0.3GCD group among the green coffee bean extract supplemented groups. The staining data showed that the average adipocyte diameter was significantly smaller in the 0.3GCD and CQD groups when compared with the HFD group (Figures 2(f) and 2(g)).

### 3.3. Plasma Biochemistries

**Figure 3:**
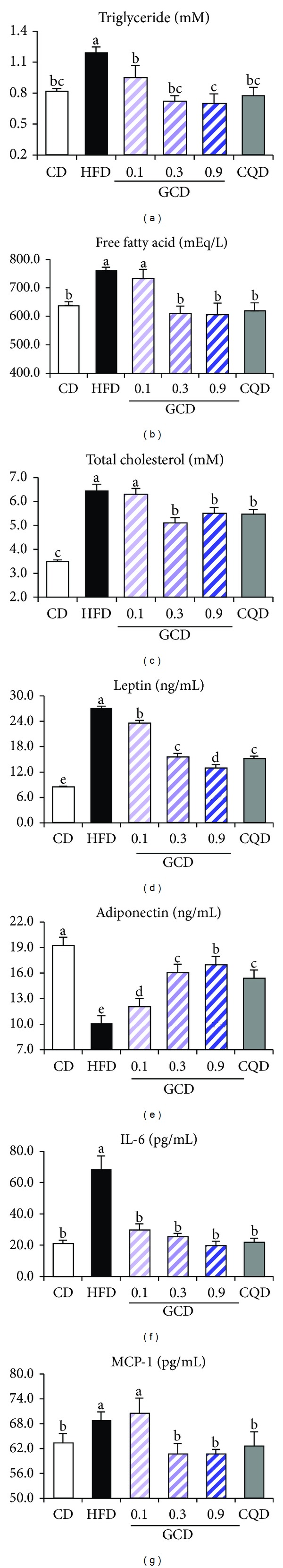
Effects of green coffee bean extract and 5-CQA supplementation on plasma levels of lipids, leptin, adiponectin, and proinflammatory cytokines in mice fed a HFD. (a) TG, (b) FFA, (c) TC, (d) leptin, (e) adiponectin, (f) IL-6, and (g) MCP-1. Bars represent mean ± SEM, *n* = 8. Mean values indicated with different letters indicate statistical significance (*P* < 0.05).

Mice fed a 0.3GCD exhibited significantly lower levels of plasma lipids, leptin, and cytokines and significantly higher levels of adiponectin than those fed a HFD (Figure 3). Yet, mice fed a 0.9GCD showed no further decreases in these plasma measures. The changes in plasma biomarkers related to obesity and inflammation that occurred in the 0.3GCD group were similar to those observed with the CQD group.

### 3.4. Glucose Utilization and Insulin Sensitivity

**Figure 4:**
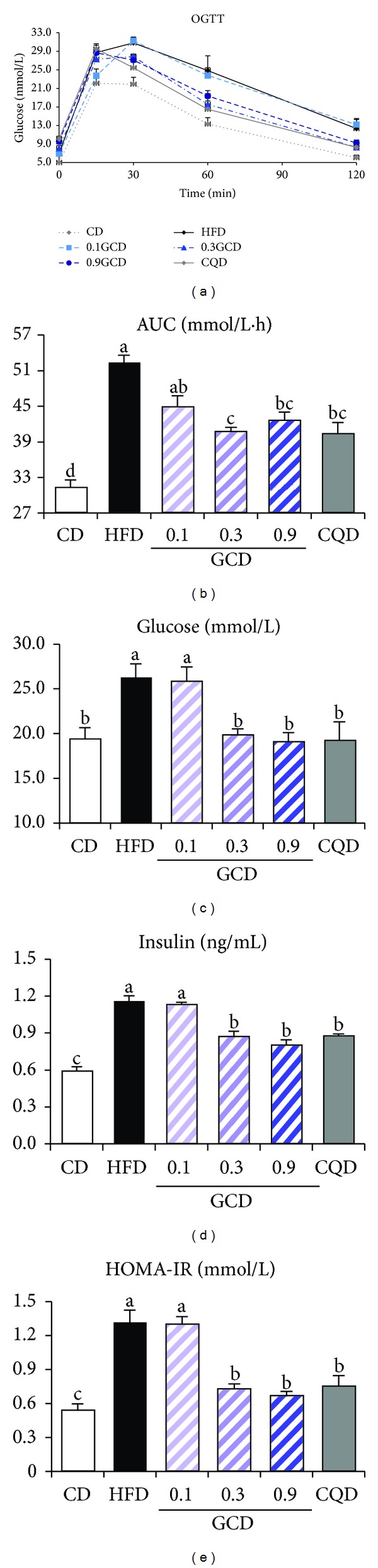
Effect of green coffee bean extract and 5-CQA supplementation on glucose utilization and insulin sensitivity in mice fed a HFD. (a) OGTT; (b) AUC; (c) glucose; (d) insulin; (e) HOMA-IR. Bars represent mean ± SEM, *n* = 8. Mean values indicated with different letters indicate statistical significance (*P* < 0.05).

We investigated whether green coffee bean extract influenced insulin sensitivity. OGTT was performed after 9 weeks of diet supplementation to determine the effect of green coffee bean extract and 5-CQA on glucose tolerance in HFD-fed mice (Figure 4(a)). Integrated plasma glucose concentration, as calculated by the area under the curve (AUC), was significantly reduced in 0.3GCD-fed mice than in HFD-fed mice (Figure 4(b)). Moreover, 0.3GCD-fed mice demonstrated a similar reduction level in the integrated glucose concentration as CQD-fed mice did. Furthermore, when fasting plasma glucose and insulin levels were measured at the end of the feeding period, 0.3GCD group exhibited significant reductions in plasma glucose and insulin levels in comparison to the HFD group (Figures 4(c) and 2(d)). Along with the plasma glucose and insulin levels, the HOMA-IR values indicated that insulin sensitivity was improved significantly in 0.3GCD group (Figure 4(e)). No further decreases in these markers related to glucose utilization and insulin sensitivity were shown in 0.9GCD-fed mice. The effect of 0.3GCD to decrease plasma glucose and insulin levels and to improve insulin sensitivity was quantitatively similar to that of CQD.

### 3.5. Expression of Genes Related to Adipogenesis

**Figure 5:**
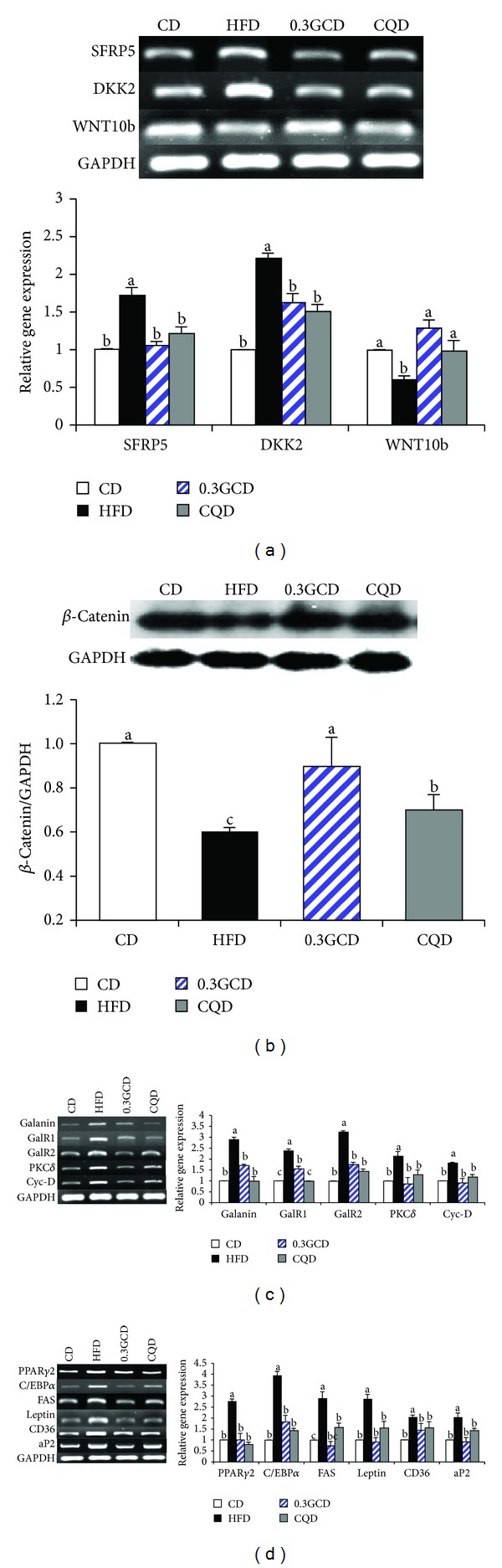
Effects of green coffee bean extract and 5-CQA supplementation on genes regulating adipogenesis in mice fed the HFD. (a) The expression of WNT10b-related genes in the epididymal adipose tissue was determined by RT-PCR and normalized to that of GAPDH. (b) Protein levels of *β*-catenin and GAPDH in the epididymal adipose tissue were determined by Western blotting. (c) and (d) The expression of galanin-related and adipogenic target genes in the epididymal adipose tissue was determined by RT-PCR and normalized to that of GAPDH. Data represent the results of three independent experiments (*n* = 2, 3 mice per experiment). *P* < 0.05 indicates statistical significance. Values are the mean ± SEM, *n* = 8 for each group.

We explored the potential mechanisms by which green coffee bean extract may attenuate HFD-induced adipogenesis in the epididymal adipose tissue of 0.3GCD-fed mice. We found that green coffee bean extract decreased the expression of secreted frizzled-receptor protein 5 (SFRP5) and dickkopf 2 (DKK2) and increased expression of wingless-type MMTV integration site family 10b (WNT10b) (Figure 5(a)). Western blot analysis indicated that *β*-catenin levels were significantly elevated by green coffee bean extract supplementation (Figure 5(b)). Furthermore, green coffee bean extract significantly reduced mRNA levels of galanin, galanin receptor (GalR) 1, GalR2, protein kinase C (PKC) *δ*, and cyclin-D (Figure 5(c)). As Figure 5(d) demonstrates, the mRNA levels of adipogenic transcription factors, PPAR*γ*2 and C/EBP*α*, were significantly downregulated in mice fed the green coffee bean extract compared with those in mice fed the HFD. The mRNA levels of key adipogenic genes such as FAS, leptin, CD36, and aP2 were significantly decreased in green coffee bean extract supplemented groups (Figure 5(d)). Moreover, these significant changes in the expression of adipogenic genes that occurred in the 0.3GCD group were similar to those observed in the CQD group (Figure 5(d)).

### 3.6. Expression of Inflammation-Related Signaling Molecules

**Figure 6:**
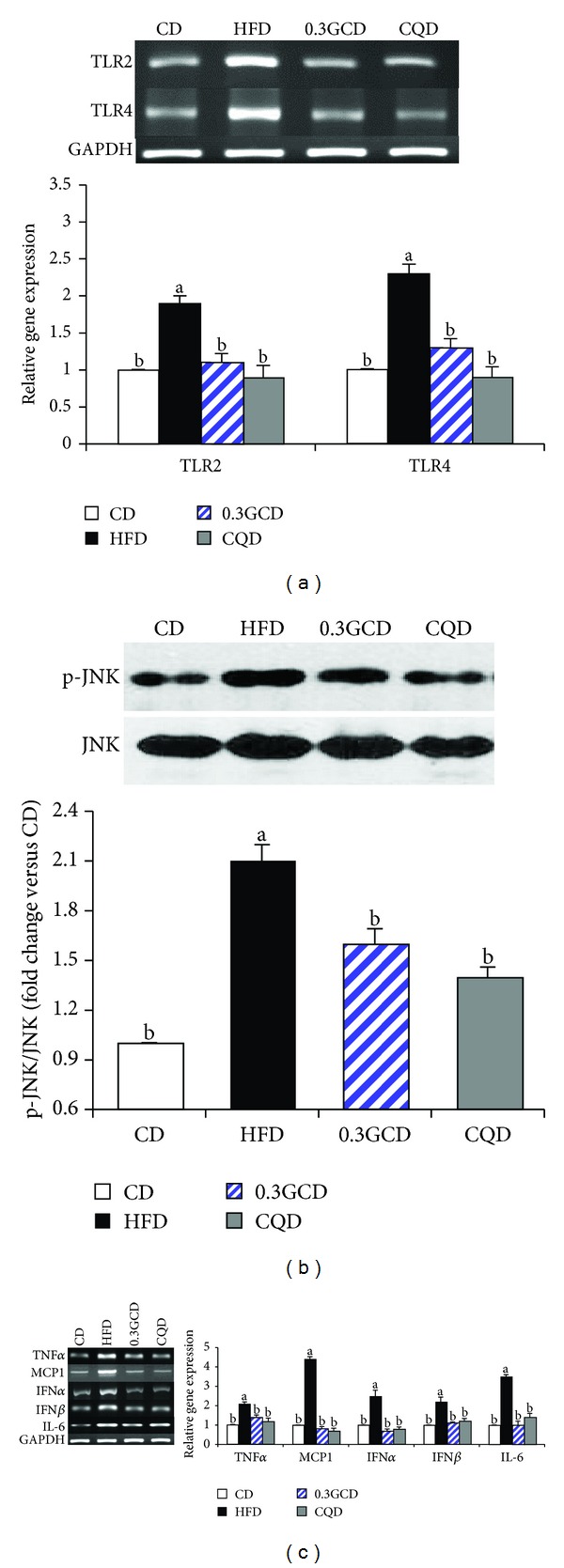
Effects of green coffee bean extract and 5-CQA supplementation on the expression of gene involved in inflammation in mice fed a HFD. (a) The expression of TLR2 and TLR4 in epididymal adipose tissue was determined by RT-PCR and normalized to that of GAPDH. (b) Protein levels of p-JNK and JNK in the epididymal adipose tissue were determined by Western blotting. (c) The expression of proinflammatory cytokine genes in the epididymal adipose tissue was determined by RT-PCR and normalized to that of GAPDH. Data represent the results of three independent experiments (*n* = 2, 3 mice per experiment). *P* < 0.05 indicates statistical significance. Values are the mean ± SEM, *n* = 8 for each group.

We examined the anti-inflammatory effects of green coffee bean extract in the epididymal adipose tissue of mice maintained on the HFD. Gene expression of inflammatory mediators such as TLR2 and TLR4 was downregulated in 0.3GCD-fed mice when compared with HFD-fed mice (Figure 6(a)). Furthermore, immunoblot results indicated that JNK phosphorylation in GCD-fed mice occurred significantly less than in HFD-fed mice (Figure 6(b)). The expression of proinflammatory cytokines, including tumor necrosis factor (TNF) *α*, MCP-1, interferon (IFN) *α*, IFN*β*, and IL-6, in GCD-fed mice was significantly downregulated in comparison with those in HFD-fed mice (Figure 6(c)). These significant changes of gene expression related to inflammation exhibited in 0.3GCD-fed mice were also shown in CQD-fed mice (Figure 6).

### 3.7. Expression of Genes Related to Insulin Resistance

**Figure 7:**
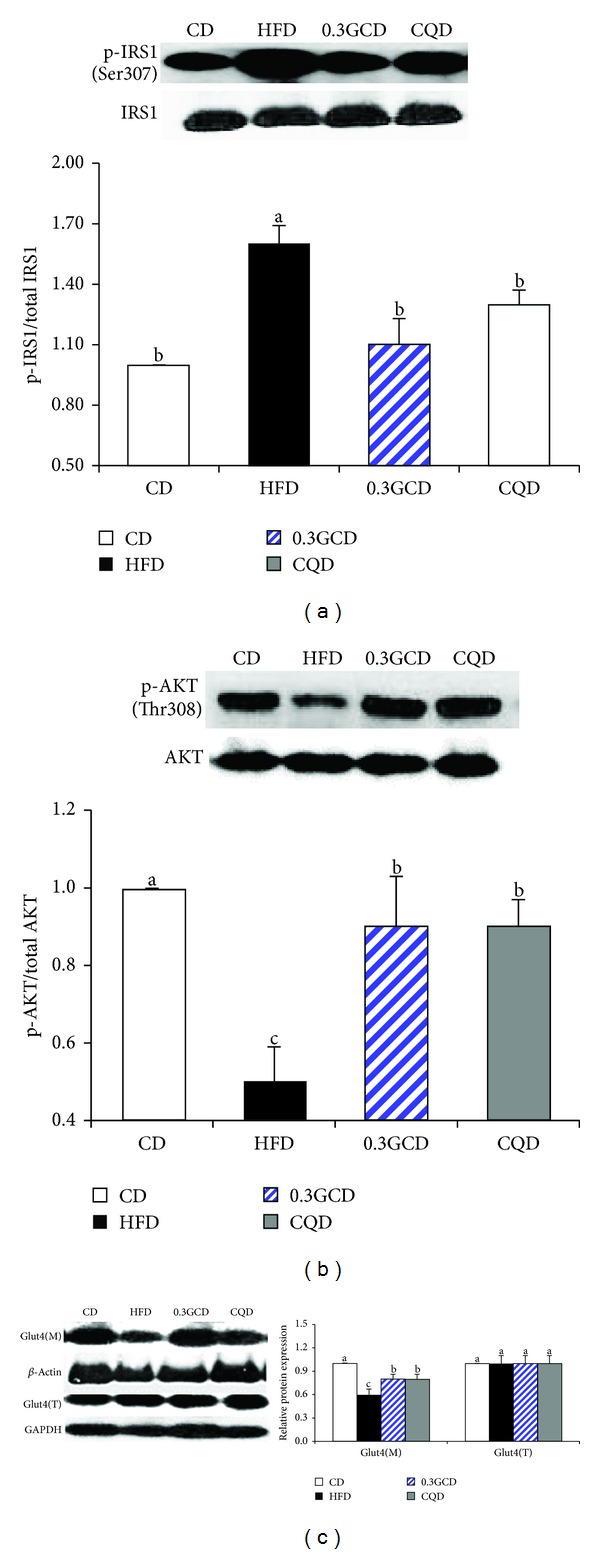
Effects of green coffee bean extract and 5-CQA supplementation on the proteins involved in GLUT4 translocation in mice fed a HFD. Protein levels of p-IRS1, p-AKT, plasma membrane GLUT4, and corresponding total proteins in the epididymal adipose tissue were determined by Western blotting. Data represent the results of three independent experiments (*n* = 2, 3 mice per experiment). *P* < 0.05 indicates statistical significance. Values are the mean ± SEM, *n* = 8 for each group.

We evaluated the protein levels of genes involved in insulin resistance in the epididymal fat tissue of 0.3GCD-fed mice. Western blot analysis revealed that phosphorylation of IRS-1 (Ser307) and AKT (Thr308) was significantly decreased and increased, respectively, in GCD-fed mice when compared to HFD-fed mice (Figures 7(a) and 7(b)). The amount of GLUT4 found in the membrane fraction of the epididymal adipocytes was significantly increased, whereas the amount of total cellular GLUT4 in cytosol remained the same in green coffee bean extract-fed mice when compared with HFD-fed mice (Figure 7(c)). Moreover, 0.3GCD was as effective in improving insulin sensitivity and enhancing GLUT4 translocation to the membrane as CQD was (Figure 7).

## 4. Discussion

In the present study, decaffeinated green coffee bean extract has demonstrated a significant weight-lowering effect in HFD-fed mice. Among the green coffee bean extract dosages (0.1%, 0.3%, and 0.9%), 0.3% green coffee bean extract was proved to be the minimum effective dose for preventing body weight gain, fat accumulation, and insulin resistance in mice fed the HFD for 11 weeks. No further dose-dependent decreases in body weight gain, visceral fat-pad weights, and plasma lipids and glucose profiles were noted at 0.9% green coffee bean extract dosage. The dose of 0.3% green coffee bean extract (300 mg green coffee bean extract/kg diet) in mice corresponds to approximately 1,460 mg/60 kg body weight in human when calculated on the basis of normalization to body surface area as recommended by Reagan-Shaw et al. [20]. In order to obtain 1.460 mg of decaffeinated green coffee bean extract, 9.7 grams of decaffeinated green coffee beans is required as calculated from its extraction yield of 15%. According to Moon, the content of total chlorogenic acids is reduced by approximately 90% in dark roasted beans [18]. This implies that 10 times more dark roasted beans (97 grams) are required to produce similar weight-reducing effects as the decaffeinated green coffee beans.

Green coffee beans are a rich source of polyphenols, especially chlorogenic acids. Of a variety of chlorogenic acids, 5-CQA has been known to protect tissues from oxidative stress, modulate glucose metabolism, and mediate antiobesity effect [12, 21, 22]. In the present study, 5-CQA was the most abundant and active component contained in green coffee bean extract and exerted a significant weight-lowering effect in HFD-fed mice (Figure 1). Based on our assumption that the antiobesity effect of decaffeinated green coffee bean extract was expected to be dose dependent and be derived from 5-CQA, the dose of 5-CQA in CQD was chosen to match the amount of 5-CQA contained in the 0.9GCD (the highest dose of decaffeinated green coffee bean extract). Nevertheless, weight-suppressing and insulin-sensitizing effects of green coffee bean extract plateaued at 0.3% supplementation. The 0.3GCD (containing 0.05% 5-CQA) significantly reduced body weight gain and improved insulin sensitivity as much as the CQD (containing 0.15% 5-CQA) did. This effect could be possibly due to other polyphenols contained in green coffee bean extract which exert beneficial effects against obesity and insulin resistance. The extent to which 0.3GCD decreased body weight gain and increased insulin sensitivity in mice fed the HFD was also shown in 0.9GCD. Exhibiting no further preventive effect against obesity and insulin resistance in 0.9GCD, green coffee bean extract seems to reach its maximum effect at 31% and 24% reductions in body weight gain and fasting plasma glucose, respectively, in HFD-fed mice.

**Figure 8:**
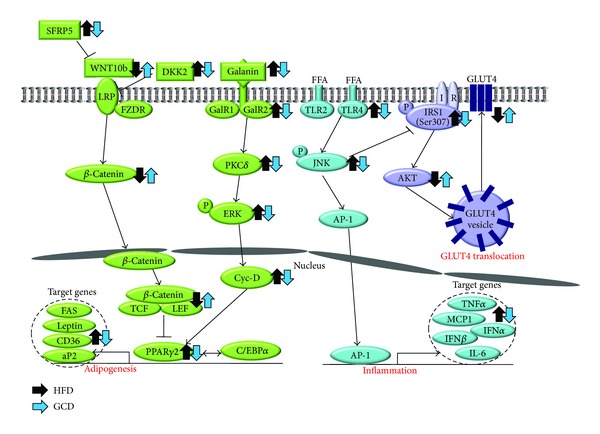
The possible molecular mechanisms of decaffeinated green coffee bean extract in attenuating adipogenesis, inflammation, and insulin resistance induced by HFD. Decaffeinated green coffee bean extract reverses HFD-induced changes in expression of genes involved in WNT10b- and galanin-mediated adipogenesis cascades in the epididymal adipose tissue. The downstream adipogenic transcription factors (PPAR*γ*2 and C/EBP*α*) and their target genes were also suppressed by decaffeinated green coffee bean extract in the epididymal adipose tissue. Decaffeinated green coffee bean extract reverses the HFD-induced changes in the expression of genes related to TLR2/4-mediated proinflammatory signaling cascades and proteins involved in GLUT4 translocation in the epididymal adipose tissue.

As food intake did not differ among the experimental groups, we can hypothesize that the weight-reducing effect of green coffee bean extract is mediated by the inhibition of adipogenesis. Indeed, we have confirmed from mRNA expression levels that adipogenic target genes of PPAR*γ*2 and C/EBP*α* were downregulated in mice fed the green coffee bean extract. Several upstream molecules such as WNT10b, galanin, fibroblast growth factor 1, and bone morphogenetic proteins induce PPAR*γ*2 and C/EBP*α* [23–25]. Of the various upstream molecules, the adipogenic mechanism of WNT10b has been thoroughly researched both* in vivo* and* in vitro *[23]. WNT signaling initiates as WNT10b, a glycoprotein, binds to fizzled receptors (FZDR) and lipoprotein-receptor-related protein (LRP) [23]. Upon WNT10b signaling activation, *β*-catenin phosphorylation and its subsequent degradation are prevented [26]. Then, hypophosphorylated *β*-catenin accumulates in the cytoplasm and translocates into the nucleus, where it binds to the T-cell-specific transcription factor/lymphoid-enhancer-binding factor (TCF/LEF) and suppresses PPAR*γ*2 and C/EBP*α*. The WNT10b signaling pathway is suppressed by extracellular antagonists, such as SFRP5 and DKK2. Secreted from adipocytes, SFRP5 and DKK2 inhibit WNT10b signaling in adipose tissue via an autocrine/paracrine mechanism (Figure 8). SFRP5 binds and sequesters WNT10b from FZD receptors, whereas DKK2 binds and disrupts LRP from binding to FZDR [27, 28]. Mice fed the green coffee bean extract exhibited lower gene expression of SFRP5 and DKK2 compared with those fed the HFD only. Transcriptional regulation on SFRP5 and DKK2 genes has not yet been explored in WAT. Yet, Qi et al. have reported that epigenetic silencing of SFRP5 mediated by hypermethylation causes constitutive activation of WNT signaling and colorectal tumorigenesis in colorectal tumor cells [29]. Epigenetic regulation of the SFRP5 gene in adipose tissue has not been reported; however, similar epigenetic silencing of the SFRP5 gene could possibly induce WNT signaling in adipose tissue. Perhaps, green coffee bean extract plays a role in epigenetic silencing of SFRP5, resulting in lower SFRP5 expression in adipose tissue but whether it directly or indirectly modulates its expression requires further investigation.

Galanin, a 29/30-residue neuropeptide, regulates food consumption, memory, neurogenesis, and neuroendocrine function by binding to receptors in the hypothalamic regions [30]. It is well known that galanin is expressed and widely distributed in the central nervous system. However, recent studies have revealed that galanin expression has also been observed in peripheral tissues such as stomach, WAT, and taste buds [24, 31]. In our previous study, we discovered that diet-induced obese mice exhibited the upregulation of galanin and its receptors in WAT [24]. As a result, increased levels of galanin activate galanin receptor subtypes, which are members of the G protein-coupled receptor family. Signaling properties of GalR1 and GalR2 are somewhat different, but both eventually lead to the activation of ERK. Upon prolonged HFD consumption, the activation of GalR1 increases mitogen-activated protein kinase (MAPK) activity in a PKC-independent manner by coupling to Gi-type G-proteins via G*βγ* subunits and induces extracellular signal-regulated kinases 1/2 (ERK) activation [32]. The activation of GalR2, coupled to Gq/11-type G-proteins, increases phospholipase C (PLC) activation. PLC cleaves phosphatidylinositol 4,5-bisphsphate into inositol 1,4,5-triphosphate and diacyl glycerol, a PKC*δ* activator. Subsequently, PKC*δ* induces the activation of ERK, which increases the expression of PPAR*γ*2 and C/EBP*α* and promotes adipogenesis. The mRNA levels of genes involved in galanin-mediated adipogenesis were reduced in mice fed green coffee bean extract. Green coffee bean extract appears to suppress adipogenesis by reversing HFD-induced increased expression of galanin, its receptors, and adipogenic transcription factors.

Since leptin secretion from adipose tissues is positively correlated with TG accumulation in adipocytes, plasma leptin level is a biomarker in assessing obesity in both experimental animals and humans [33, 34]. Increments of serum leptin levels usually occur together with adipocyte hypertrophy. In the present study, HFD-fed mice demonstrated higher plasma leptin levels and larger adipocyte size than CD-fed mice. Yet, green coffee bean extract seems to decrease plasma leptin and its mRNA expression along with the average adipocyte diameter in HFD-fed mice.

Lower levels of plasma proinflammatory cytokines such as IL-6 and MCP-1 were observed in mice fed green coffee bean extract when compared with mice fed the HFD. Generally, the levels of plasma cytokines correlate with the expression of proinflammatory adipokines in WAT [35, 36]. Adipocytes under normal or healthy conditions referred to as “lean” fat cells engage metabolic pathways, leaving immune-response pathways inactive [36]. However, when “lean” fat cells are constantly exposed to overnutrition and store excess fat beyond a “critical size,” they become “fat” fat cells, which are a good source of adipokines [37]. In this modified milieu, from a metabolic to proinflammatory environment, inflammatory mediators such as TLR2 and TLR4 are chronically expressed in adipocytes [36]. TLRs play a role in the innate immune response, which discriminates between “self” and “nonself” and activates proinflammatory processes upon stimulation via pathogen-associated molecular patterns such as lipopolysaccharides [38]. Activation of TLRs induces phosphorylation of JNK and recruitment of activator protein-1 (AP-1), which acts as a transcriptional activator of proinflammatory cytokines such as TNF*α*, MCP1, IFN*α*, IFN*β*, and IL-6 [16, 39–41]. Our data revealed that the expression of TLR2 and TLR4 was reduced in mice fed the green coffee bean extract when compared with those fed the HFD. This implies that green coffee bean extract may attenuate WAT from becoming a HFD-induced inflamed and modified environment. Recently, it was reported that fatty acids, particularly saturated fatty acids (C14:0, C16:0, and C18:0), serve as ligands to TLR4 [16, 40]. In the present study, mice fed the green coffee bean extract exhibited lower plasma FFA levels when compared with those fed a HFD. From this, we suggest that green coffee bean extract may have attenuated activation of TLR4-mediated signaling pathway and therefore resulted in the decreased expression of proinflammatory cytokines, confirmed by their mRNA expression levels. Collectively, we speculate that green coffee bean extract may suppress proinflammatory responses by not only decreasing the expression of TLR2 and TLR4 but also decreasing ligand-induced TLR4-mediated inflammatory signaling.

Fasting plasma glucose and insulin levels and OGTT results demonstrated that insulin sensitivity was enhanced in green coffee bean extract fed mice. Improvement in insulin sensitivity was further confirmed by Western blot analysis of insulin signaling-related molecules and membrane GLUT4. Mice fed the green coffee bean extract exhibited increased phosphorylation of AKT and translocation of GLUT4 from the cytosol to the membrane when compared with those fed a HFD. Whether green coffee bean extract has a direct influence on GLUT4 translocation requires further investigation. However, we speculate that the beneficial effects against insulin resistance could be associated with decreased inflammatory responses. Recent studies have suggested that JNK, an important stress sensor, plays a crucial role in the regulation of HFD-induced insulin resistance and inflammation [36]. When WAT becomes inflamed, JNK is phosphorylated and p-JNK inactivates IRS-1 by phosphorylating its serine residue [39]. Serine phosphorylation on IRS-1 blocks GLUT4 translocation and therefore impairs insulin sensitivity [42]. Thus, green coffee bean extract may have reversed HFD-induced insulin resistance by decreasing JNK activation and increasing GLUT4 translocation.

In conclusion, the present study shows that green coffee bean extract significantly reduces visceral fat-pad accumulation and improves insulin resistance in mice fed the HFD at a minimum effective dose of 0.3% supplementation. We suggest that 5-CQA and other polyphenols in green coffee bean extract may bring an additive effect in decreasing body weight gain and increasing insulin sensitivity. These beneficial effects are possibly due to the downregulation of genes associated with adipogenesis and inflammation in the WAT of mice. Taken as a whole, decaffeinated green coffee bean extract may be used as a therapeutic agent that prevents obesity and metabolic syndrome.

## Figures and Tables

**Table 1:** Primer sequences and PCR conditions.

Gene description	Primers	Sequences (5′-3′)	*T*_*m*_ (°C)	Size (bp)
SFRP 5	F	CTTGGTGTCCTTGCGCTTTA	61	155
	R	CTGATGGCCTCATGGAACAG		
DKK2	F	TCATTCCCTGTTCTTCAGCG	55	144
	R	GCATTTCCTTCAGATTGGCA		
WNT10b	F	TTTTGGCCACTCCTCTTCCT	61	183
	R	TCCTTTTCCAACCGAAAACC		
Galanin	F	GAGCCTTGATCCTGCACTGA	60	121
	R	AGTGGCTGACAGGGTCACAA		
GalR1	F	CCAAGGGGGTATCCCAGTAA	60	147
	R	GGCCAAACACTACCAGCGTA		
GalR2	F	ATAGTGGTGCTCATGCTGGAA	60	134
	R	AGGCTGGATCGAGGGTTCTA		
PKC*δ*	F	CTGAGCGCTGCAAGAAGAAC	60	146
	R	TGGAAACTTTGATCCTGCACTGA		
Cyc-D	F	TGGGAAGTTTTGTTGGGTCA	55	144
	R	TCCTTGTCCAGGTAATGCCA		
PPAR*γ*2	F	TTCGGAATCAGCTCTGTGGA	55	148
	R	CCATTGGGTCAGCTCTTGTG		
C/EBP*α*	F	AAGGCCAAGAAGTCGGTGGA	55	189
	R	CCATAGTGGAAGCCTGATGC		
FAS	F	TTGCCCGAGTCAGAGAACC	55	171
	R	CGTCCACAATAGCTTCATAGC		
Leptin	F	CTCCAAGGTTGTCCAGGGTT	55	143
	R	AAAACTCCCCACACAATGGG		
CD36	F	ATGACGTGGCAAAGAACAGC	55	160
	R	GAAGGCTCAAAGATGCCTCC		
aP2	F	ACATGAAAGTGGGAGTG	55	128
	R	AAGTACTCTCTGACCGGATG		
TLR2	F	TCTAAAGTCGATCCGCGACAT	55	344
	R	TACCCAGCTCGCTCACTACGT		
TLR4	F	ACCTCTGCCTTCACTACAGA	48.6	223
	R	AGGGACTTCTCAACCTTCTC		
TNF*α*	F	TGTCTCAGCCTCTTCTCATT	55	156
	R	AGATGATCTGAGTGTGAGGG		
MCP1	F	CCAGCAAGATGATCCCAATG	55	450
	R	CTTCTTGGGGTCAGCACAGA		
IFN*α*	F	ATGGCTAGGCTCTGTGCTTTCCT	58	638
	R	GGGCTCTCCAGATTTCTGCTCTG		
IFN*β*	F	CCACAGCCCTCTCCATCAACTATAAGC	56	372
	R	AGCTCTTCAACTGGAGAGCAGTTGAGG		
IL-6	F	TTGCCTTCTTGGGACTGATG	55	162
	R	CCACGATTTCCCAGAGAACA		
GAPDH	F	AGAACATCATCCCTGCATCC	60	321
	R	TCCACCACCCTGTTGCTGTA		

SFRP 5: secreted frizzled-related protein 5; DKK2: Dickkopf 2; WNT10b: wingless-type MMTV integration site family, member 10B; GalR1: galanin receptor 1; PKC*δ*: protein kinase C delta; Cyc-D: cyclin D; PPAR*γ*2: peroxisome proliferator-activated receptor gamma; C/EBP*α*: CCAAT/enhancer-binding protein, alpha; FAS: fatty acid synthase; CD36: fatty acid translocase; aP2: adipocyte protein2; TLR: Toll-like receptor; TNF*α*: tumor necrosis factor alpha; IFN: interferon; MCP1: monocyte chemoattractant protein 1; IL-6: interleukin-6; GAPDH: gyceraldehyde-3-phosphate dehydrogenase.
